# T7 RNA polymerase‐driven inducible cell lysis for DNA transfer from *Escherichia coli* to *Bacillus subtilis*

**DOI:** 10.1111/1751-7915.12843

**Published:** 2017-08-16

**Authors:** Mario Juhas, James W. Ajioka

**Affiliations:** ^1^ Department of Pathology University of Cambridge Tennis Court Road CB2 1QP Cambridge UK

## Abstract

The majority of the good DNA editing techniques have been developed in *Escherichia coli*; however, *Bacillus subtilis* is better host for a plethora of synthetic biology and biotechnology applications. Reliable and efficient systems for the transfer of synthetic DNA between *E. coli* and *B. subtilis* are therefore of the highest importance. Using synthetic biology approaches, such as streamlined lambda Red recombineering and Gibson Isothermal Assembly, we integrated genetic circuits *pT7*L123, Repr‐ts‐1 and *pL*T7pol encoding the lysis genes of bacteriophages MS2, ΦX174 and lambda, the thermosensitive repressor and the T7 RNA polymerase into the *E. coli* chromosome. In this system, T7 RNA polymerase regulated by the thermosensitive repressor drives the expression of the phage lysis genes. We showed that T7 RNA polymerase significantly increases efficiency of cell lysis and transfer of the plasmid and bacterial artificial chromosome‐encoded DNA from the lysed *E. coli* into *B. subtilis*. The T7 RNA polymerase‐driven inducible cell lysis system is suitable for the efficient cell lysis and transfer of the DNA engineered in *E. coli* to other naturally competent hosts, such as *B. subtilis*.

## Introduction

The Gram‐negative bacterium *Escherichia coli* and the Gram‐positive bacterium *Bacillus subtilis* are among the key cellular chassis suitable for a plethora of synthetic biology devices and biotechnology applications (Harwood and Cranenburgh, 2008; Ajikumar *et al*., 2010; Commichau *et al*., 2014; Juhas and Ajioka, 2016). Consequently, both *E. coli* and *B. subtilis* have been successfully used for the production of a number of the industrially important products, such as biofuels, biosensors, pharmaceuticals and natural dyes (Ajikumar *et al*., 2010; Yim *et al*., 2011; Park *et al*., 2012; Zhou *et al*., 2012; Hao *et al*., 2013; Manabe *et al*., 2013; McKenney *et al*., 2013). Furthermore, both *E. coli* and *B. subtilis* are considered to be promising chassis for engineering of the minimal and tailor‐made cell factories (Commichau *et al*., 2013; Juhas *et al*., 2014b; Juhas, 2015; Juhas and Ajioka, 2016). The majority of the good DNA editing techniques have been developed in *E. coli*; however, *B. subtilis* has many advantages. This includes formation of endospores able to withstand severe environmental insults and secretion of the industrially valuable proteins into the cultivation medium. Furthermore, natural competence allows easy transformation of *B. subtilis* with foreign DNA and its integration into the chromosome by homologous recombination (Shi *et al*., 2013; Yadav *et al*., 2014).

The reliable and efficient systems for transfer of synthetic DNA, particularly of the high‐molecular‐weight DNA, which is prone to physical breakage, between *E. coli* and *B. subtilis* chassis are therefore of the highest importance. Several methods have been developed to address this issue in the last few years. The conditionally inducible cell lysis system relying on the lambda prophage has been used successfully for the transfer of DNA released from the lysed *E. coli* cells into *B. subtilis* (Itaya and Kaneko, 2010; Kaneko and Itaya, 2010; Gao *et al*., 2013). In this system, the co‐culture method was used to transfer the high‐molecular‐weight DNA from *E. coli* undergoing lambda prophage‐induced lysis to *B. subtilis* (Itaya and Kaneko, 2010; Kaneko and Itaya, 2010). Furthermore, transfer of genetic circuits from the lysed *E. coli* to *B. subtilis*, which does not rely on the entire phage to lyse the donor cell, has been demonstrated recently (Juhas *et al*., 2016). This system utilizes only the individual lysis genes from multiple phages to disrupt the donor *E. coli* cell. A similar system, based on the lytic genes of the lactococcal bacteriophage ΦUS3, has been developed for an inducible lysis of *Lactococcus lactis* for accelerated cheese ripening (de Ruyter *et al*., 1996, 1997).

T7 RNA polymerase expression system, based on the RNA polymerase of bacteriophage T7, is among the most efficient and widely used systems for the expression of recombinant proteins (Studier and Moffatt, 1986; Kortmann *et al*., 2015; Wei *et al*., 2017). T7 RNA polymerase is independent of the host auxiliary transcription factors, has high processivity and specificity towards the *pT7* promoter, and is able to produce long transcripts faster than the multi‐subunit bacterial RNA polymerase (Kortmann *et al*., 2015; Wei *et al*., 2017). Consequently, T7 polymerase has been successfully used for regulating protein expression in a number of bacteria, including *Pseudomonas aeruginosa, Pseudomonas putida, Streptomyces lividans, Streptomyces coelicolor, Rhodobacter capsulatus, Ralstonia eutropha, Bacillus megaterium*,* Corynebacterium acetoacidophilum* and *Corynebacterium glutamicum* (Brunschwig and Darzins, 1992; Herrero *et al*., 1993; Barnard *et al*., 2004; Gamer *et al*., 2009; Lussier *et al*., 2010; Arvani *et al*., 2012; Equbal *et al*., 2013; Kortmann *et al*., 2015; Wei *et al*., 2017).

Here, we present an inducible cell lysis system, which utilizes the chromosomally integrated genetic circuit encoding T7 RNA polymerase, to drive the expression of the phage lysis genes for the reliable and efficient cell lysis and plasmid and bacterial artificial chromosome (BAC)‐borne DNA transfer from *E. coli* into *B. subtilis*.

## Results and discussion

### Construction of the T7 RNA polymerase‐driven inducible cell lysis system

**Figure 1:**
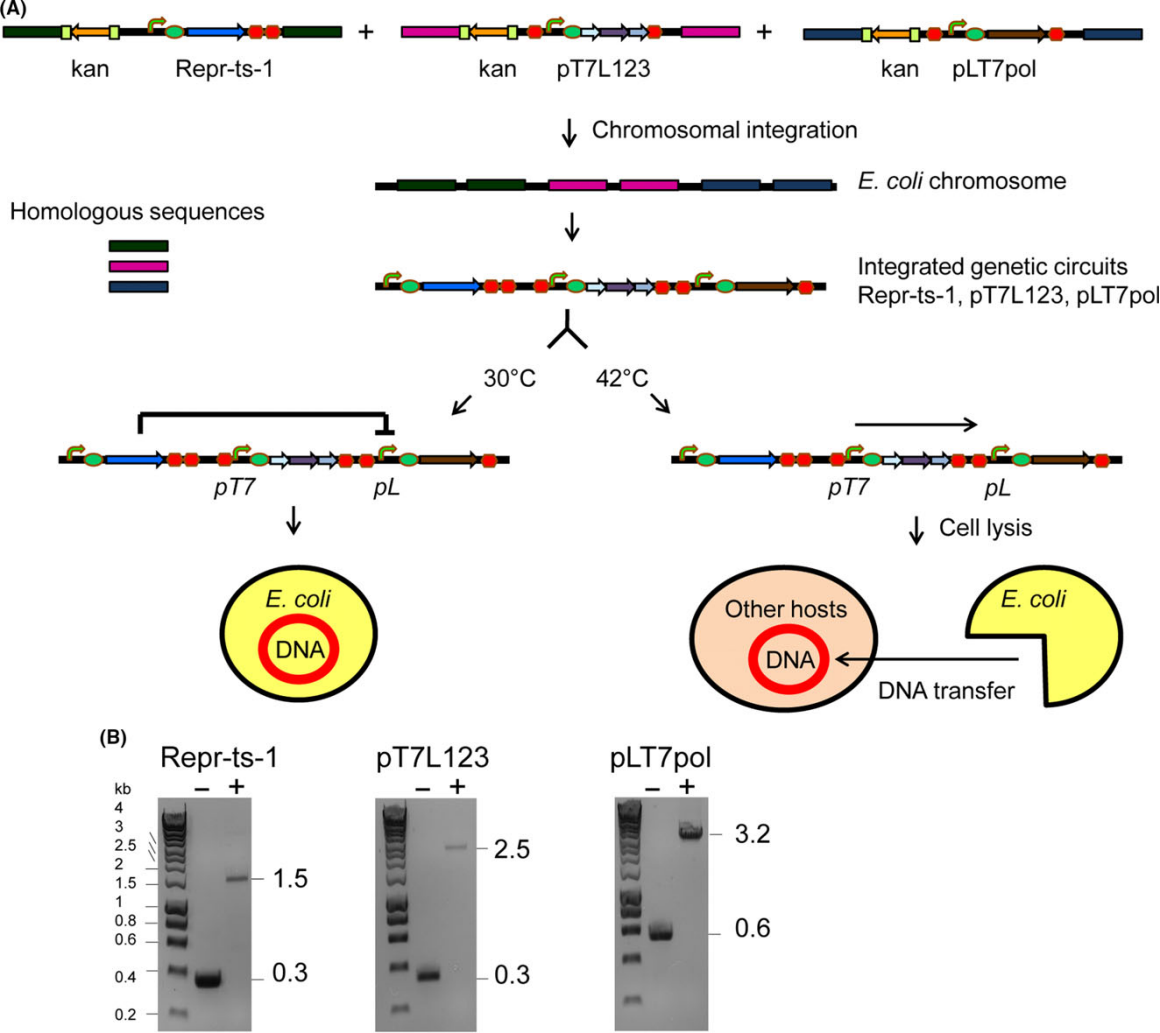
The T7 RNA polymerase‐driven temperature‐inducible cell lysis system. A. Figure shows construction of the T7 RNA polymerase‐driven inducible *Escherichia coli* cell lysis system. The genetic circuits *pT7*L123, Repr‐ts‐1 and *pL*T7pol encoding the phage MS2, ΦX174 and lambda lysis genes, the thermosensitive repressor and the T7 RNA polymerase, respectively, were integrated into the *E. coli* chromosome using the streamlined lambda Red recombinase‐mediated method. The kanamycin resistance cassette flanked by flippase recognition target (FRT) sites was removed from the chromosome using flippase (FLP) recombinase. At 30°C, the expression of T7 RNA polymerase from the *pL* promoter is inhibited by the repressor. Temperature shift to 42°C alleviates this repression and leads to expression of the T7 RNA polymerase, which in turn leads to expression of the phage MS2, ΦX174 and lambda lysis genes from the *pT7* promoter and cell lysis. DNA released from the lysed *E. coli* cells can be then taken up by other hosts, such as *Bacillus subtilis*. Kan: kanamycin; *pL*: promoter controlled by the thermosensitive lambda repressor; *pT7*: promoter controlled by T7 RNA polymerase. B. Figure depicts PCR confirmation of the integration of the genetic circuits Repr‐ts‐1, *pT7*L123 and *pL*T7pol into the *E. coli* chromosome. Expected amplicon sizes are shown on the right side in kbs. − (wild type without the integrated genetic circuit), + (engineered strain with the chromosomally integrated genetic circuit). HyperLadder 1 kb (Bioline) was used as the molecular weight marker.

To construct the T7 RNA polymerase‐driven inducible cell lysis system for the controlled lysis of *E. coli* and DNA transfer from the lysed *E. coli* cells to *B. subtilis*, we first engineered genetic circuits *pT7*L123, Repr‐ts‐1 and *pL*T7pol (Fig. 1A and Fig. S1). The genetic circuit *pT7*L123 encodes lysis genes of the three bacteriophages, namely MS2, ΦX174 and lambda. We chose lysis genes of the bacteriophages MS2, ΦX174 and lambda mainly because they have different mechanism of action and are all well characterized (Young, 1992; Gründling *et al*., 2001; Berry *et al*., 2008; Shulman *et al*., 2012; Tanaka and Clemons, 2012; Catalão *et al*., 2013). Furthermore, we showed previously that combining lysis genes of the bacteriophages MS2, ΦX174 and lambda in the same cell significantly improves lysis efficiency of *E. coli* cells (Juhas *et al*., 2016). Lysis genes of the bacteriophages MS2, ΦX174 and lambda in the engineered genetic circuit *pT7*L123 are located downstream of the *pT7* promoter, which is regulated by the T7 RNA polymerase. We incorporated a RBS upstream of the first lysis gene (MS2) of the genetic circuit *pT7*L123. Furthermore, we isolated the genetic circuit *pT7*L123 with the T0 transcription terminator on the 5' end and with the t1 transcriptional terminator on the 3' end obtained from the Registry of Standard Biological Parts (http://parts.igem.org/Main_Page; Juhas *et al*., 2014a; Fig. 1A and Fig. S1).

The genetic circuit Repr‐ts‐1 encodes the thermosensitive lambda repressor regulated by a strong constitutive promoter. In addition, Repr‐ts‐1 harbours a RBS and double terminator (B0015) from the Registry of Standard Biological Parts (Fig. 1A and Fig. S1).

The genetic circuit *pL*T7pol encodes T7 RNA polymerase (Wycuff and Matthews, 2000) located downstream of the *pL* promoter, which is regulated by the thermosensitive lambda repressor (Fig. 1A and Fig. S1). Furthermore, *pL*T7pol harbours a RBS and is isolated with the T0 transcription terminator on the 5' end and with the t1 transcriptional terminator on the 3' end (Fig. 1A and Fig. S1).

We first cloned all three engineered genetic circuits *pT7*L123, Repr‐ts‐1 and *pL*T7pol into plasmid pSB1K3(FRTK; Juhas *et al*., 2014a) next to the kanamycin resistance cassette, which is flanked by FRT (flippase recombinase target) sites, employing Gibson Isothermal Assembly method (Gibson *et al*., 2009; Merryman and Gibson, 2012; Data S1). Then, we integrated the genetic circuits *pT7*L123, Repr‐ts‐1 and *pL*T7pol into the *E. coli* chromosome using the streamlined lambda Red recombinase‐mediated approach described previously (Datsenko and Wanner, 2000; Juhas *et al*., 2014a). By integrating the genetic circuits *pT7*L123, Repr‐ts‐1 and *pL*T7pol into the *E. coli* chromosome, we generated the strain Ec(R*pT7*L123*pL*T7i). We removed the kanamycin resistance marker from the integrated modules *pT7*L123, Repr‐ts‐1 and *pL*T7pol in the strain Ec(R*pT7*L123*pL*T7i) using plasmid pEP‐FLP, which encodes constitutively expressed FLP recombinase (St‐Pierre *et al*., 2013). We confirmed the correct assemblies of the genetic circuits *pT7*L123, Repr‐ts‐1 and *pL*T7pol and their successful integration into the *E. coli* chromosome by diagnostic PCR (Fig. 1B and Table S1) and sequencing.

The expression of the *pL*T7pol‐encoded T7 RNA polymerase in the engineered strain Ec(R*pT7*L123*pL*T7i) is inhibited by the Repr‐ts‐1‐encoded thermosensitive repressor (Fig. 1A). T7 RNA polymerase then in turn regulates expression of the genetic circuit *pT7*L123, which encodes lysis genes of the bacteriophages MS2, ΦX174 and lambda. At 30°C, the expression of T7 RNA polymerase from the *pL* promoter in the genetic circuit *pL*T7pol is inhibited by the Repr‐ts‐1‐encoded thermosensitive repressor. A simple temperature shift to 42°C alleviates this repression and leads to activation of the T7 RNA polymerase, which in turn leads to expression of the lysis genes of the bacteriophages MS2, ΦX174 and lambda from the *pT7* promoter and cell lysis. DNA released from the lysed *E. coli* cells can be subsequently taken up by other hosts, such as *B. subtilis* (Fig. 1A).

### RNA polymerase increases lysis efficiency of the inducible cell lysis system

To test the effect of the integrated T7 RNA polymerase on the inducible cell lysis, we compared the lysis efficiency of the engineered T7 RNA polymerase‐driven temperature‐inducible cell lysis system in the strain Ec(R*pT7*L123*pL*T7i) with the strains Ec(Ri) and Ec(R*pL*L123i). The strain Ec(Ri) only harbours the genetic circuit Repr‐ts‐1 encoding the thermosensitive repressor integrated in the chromosome. The strain Ec(R*pL*L123i) has the genetic circuits Repr‐ts‐1 and *pL*L123 integrated into the chromosome, but does not harbour the genetic circuit *pL*T7pol. Consequently, as the strain Ec(R*pL*L123i) has not been augmented with the genetic circuit *pL*T7pol, the expression of the lysis genes of the bacteriophages MS2, ΦX174 and lambda in the strain Ec(R*pL*L123i) is directly regulated by the thermosensitive repressor (Juhas *et al*., 2016) instead of through T7 RNA polymerase.

**Figure 2:**
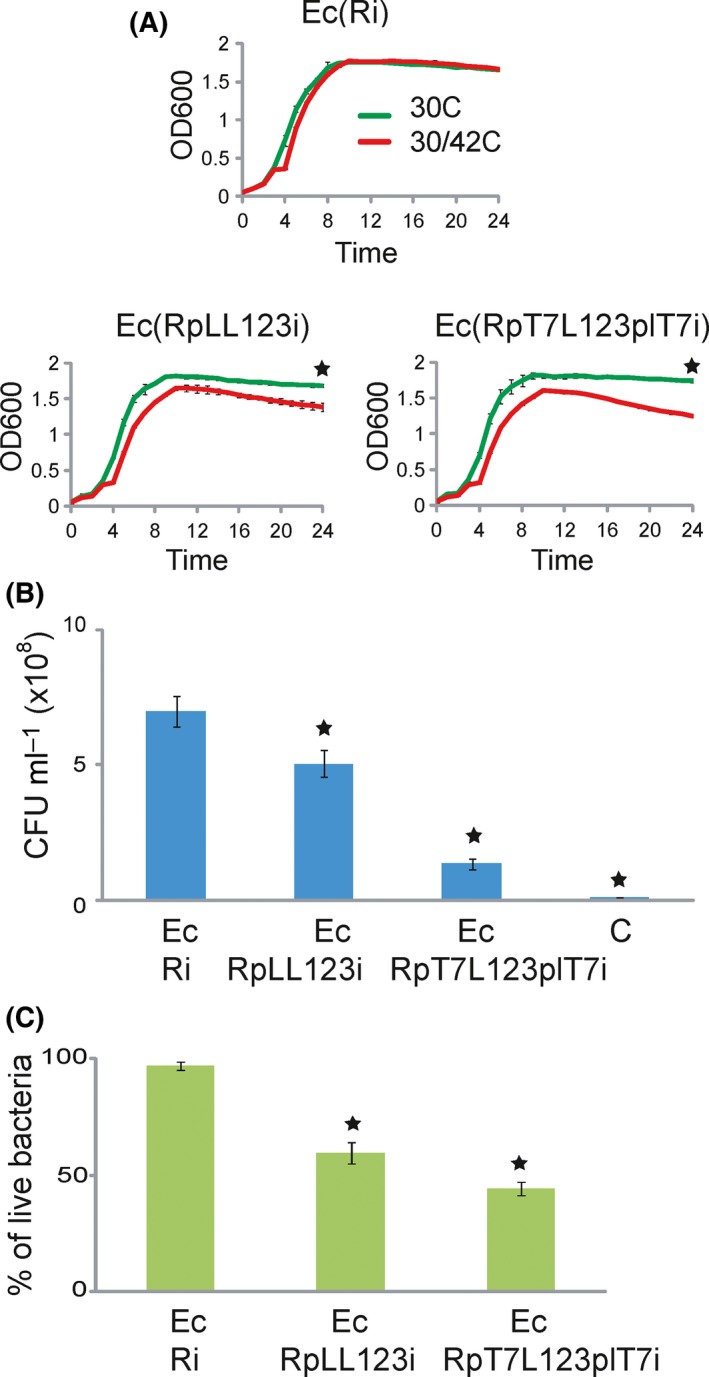
Cell lysis efficiency assays. A. Figure depicts quantification of the cell lysis measured as absorbance over time with the microplate reader for the strains Ec(Ri), Ec(R*pL*L123i) and Ec(R*pT7*L123*pL*T7i). Bacterial strains were incubated first at the restrictive conditions for the repressor for 24 h (30°C) and second at the restrictive conditions for 3 h and then at the permissive conditions for another 21 h (30/42°C). The statistical *t*‐tests were performed to compare the differences between absorbance at the restrictive and restrictive/permissive conditions for each strain at the end of the measurement period. Differences were significant for each strain with the exception of Ec(Ri). The statistical *t*‐test analysis also revealed that there was a significant difference (indicated by the star) between the strains Ec(R*pL*L123i) where the thermosensitive repressor directly controls the lysis genes and Ec(R*pT7*L123*pL*T7i) where the induction of the cell lysis is performed by cascading the induction with a T7 RNA polymerase with 11% increase in the absorbance difference. The mean values and standard deviations were calculated from the three experiments. B. Figure shows lysis efficiency for strains Ec(Ri), Ec(R*pT7*L123*pL*T7i) and Ec(R*pL*L123i) quantified from the viable CFUs per ml of each analysed strain culture 6 h after the induction of cell lysis. The lysis efficiency in strains Ec(R*pT7*L123*pL*T7i) and Ec(R*pL*L123i) was significantly lower than in the strain Ec(Ri). The statistical *t*‐test analysis performed to compare the variations in CFUs showed that the number of viable cells in the strain Ec(R*pT7*L123*pL*T7i) was significantly lower (*P* < 0.005, indicated by the star) than in the strain Ec(R*pL*L123i). C. Figure shows result of the cell viability assay by staining of the strains Ec(Ri), Ec(R*pT7*L123*pL*T7i) and Ec(R*pL*L123i) with the *Bac*Light Live/Dead bacterial viability stain. The proportion of the live/dead cells was quantified 6 h after the induction of cell lysis. The strain Ec(R*pT7*L123*pL*T7i) harbouring the chromosomally integrated T7 RNA polymerase‐encoding genetic circuit had the highest proportion of the dead cells after the temperature shift from 30°C to 42°C. The means and standard errors were calculated from the three biological replicates. The statistical *t*‐test was conducted to assess the variations in the proportion of the live/dead bacteria. Stars indicate significant difference in the proportion of the live bacteria between Ec(R*p* *L*L123i) and Ec(R*pT7*L123*p* *L*T7i).

We first analysed the cell lysis efficiency by measuring absorbance over time in the investigated strains with the microplate reader (Fig. 2A). The absorbance of the strains Ec(Ri), Ec(R*pL*L123i) and Ec(R*pT7*L123*pL*T7i) grown at the restrictive temperature for the thermosensitive repressor for 24 h was compared with the absorbance when the same strains were grown in both at the restrictive and permissive temperature (Fig. 2A). The statistical *t*‐test analysis revealed significant differences between the absorbance measured at induced and non‐induced conditions in strains Ec(R*pL*L123i; *P* < 0.005) and Ec(R*pT7*L123*pL*T7i; *P* < 0.005), but not the strain Ec(Ri). To test the effect of the T7 RNA polymerase in the strain Ec(R*pT7*L123*pL*T7i) on the cell lysis, we calculated the differences between the mean absorbances of the strains Ec(R*pT7*L123*pL*T7i) and Ec(R*pL*L123i), which does not harbour T7 RNA polymerase, grown at restrictive and restrictive and permissive conditions (Fig. 2A). The statistical *t*‐test analysis revealed significant differences between the degree of the cell lysis of Ec(R*pT7*L123*pL*T7i) and Ec(R*pL*L123i) with 11% (*P* < 0.05) increase in the differences between the mean absorbances in the strain Ec(R*pT7*L123*pL*T7i; Fig. 2A). This experiment suggested that the strain Ec(R*pT7*L123*pL*T7i) harbouring the integrated T7 polymerase‐encoding genetic circuit lysed with a significantly higher efficiency than the strain Ec(R*pL*L123i) in which the expression of the lysis genes is regulated directly by the thermosensitive repressor.

As the result of the absorbance measurement can be affected by a number of factors, such as the debris from the lysed cells, we also analysed the effect of the integrated T7 RNA polymerase on the inducible cell lysis on the level of the individual cells. To quantify the numbers of the viable cells in the strains Ec(Ri), Ec(R*pT7*L123*pL*T7i) and Ec(R*pL*L123i), we first counted the numbers of CFUs after the induction of the cell lysis as described in the Experimental procedures section (Fig. 2B). The numbers of the viable cells in both strains Ec(R*pT7*L123*pL*T7i) and Ec(R*pL*L123i) were significantly lower than in the strain Ec(Ri). Furthermore, the statistical *t*‐test analysis showed that the number of viable cells in the strain Ec(R*pT7*L123*pL*T7i) was almost four times lower (*P* < 0.005) than in the strain Ec(R*pL*L123i), which does not harbour T7 RNA polymerase, thus confirming the increased lysis efficiency of the engineered T7 RNA polymerase‐driven inducible cell lysis system (Fig. 2B).

In addition to the above experiments, we measured the cell lysis efficiency by staining the analysed strains grown at the restrictive and permissive conditions with the *Bac*Light Live/Dead bacterial viability stain and evaluated the stained bacterial strains by fluorescent microscopy. This analysis showed that the viability of strains Ec(R*pT7*L123*pL*T7i) and Ec(R*pL*L123i) was significantly lower than the viability of the strain Ec(Ri; Fig. 2C). Furthermore, the statistical *t*‐test analysis revealed a significant difference in the proportion of the live and dead bacteria in the investigated strains Ec(R*pT7*L123*pL*T7i) and Ec(R*pL*L123i) with 26% (*P* < 0.05) increase in the number of the dead bacteria and bacteria with the damaged membranes in the strain Ec(R*pT7*L123*pL*T7i) after the induction of the cell lysis (Fig. 2C).

These experiments showed that chromosomally integrated T7 RNA polymerase in the engineered T7 RNA polymerase‐driven temperature‐inducible cell lysis system in the strain Ec(R*pT7*L123*pL*T7i) significantly increases the cell lysis efficiency compared with the strain Ec(R*pL*L123i) where the cell lysis is directly controlled by the thermosensitive repressor. To compare the efficiency of the engineered T7 polymerase‐driven inducible cell lysis system with the chemical lysis system, we used a modified phenol–chloroform method (Song *et al*., 1999). To quantify the number of the viable *E. coli* cells, we counted the numbers of CFUs after the induction of the cell lysis by phenol–chloroform as described in the Experimental procedures section (Fig. 2B). The number of the viable cells after the chemical lysis was significantly lower than in strains Ec(R*pT7*L123*pL*T7i), Ec(R*pL*L123i) and Ec(Ri), thus showing that the phenol–chloroform‐induced cell lysis is more efficient than the temperature‐inducible cell lysis. Overall, the direct counting of the CFUs of the viable bacteria after the induction of the cell lysis provided the most significant difference in the lysis efficiency between the strains Ec(R*pT7*L123*pL*T7i) and Ec(R*pL*L123i), with 73% decrease in the number of the viable cells (*P* < 0.005) in the strain Ec(R*pT7*L123*pL*T7i; Fig. 2B).

### T7 polymerase‐driven inducible cell lysis for DNA transfer from *E. coli* to *B. subtilis*

We used plasmid pJScav and integrative bacterial artificial chromosome iBAC(cav)1 and iBAC(10)‐borne DNA to investigate the suitability of the T7 RNA polymerase‐driven inducible cell lysis system for transfer of DNA released from the lysed *E. coli* into *B. subtilis*. pJScav and iBAC(cav)1 harbour genetic circuits encoding *Bacillus*‐specific chloramphenicol resistance and the yellow fluorescent protein mVenus flanked by the sequences homologous to the *amyE* target site in the *B. subtilis* chromosome. In addition to chloramphenicol resistance and mVenus, iBAC(10) harbours a 10 kb DNA fragment flanked by the sequences homologous to the *amyE* site.

**Figure 3:**
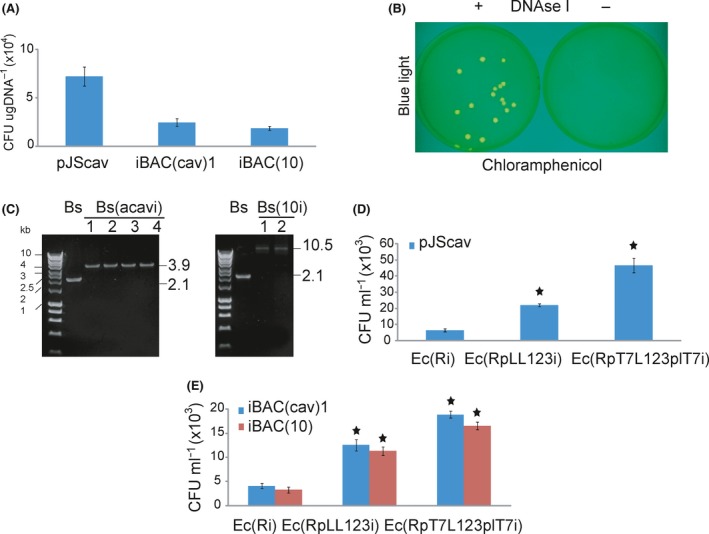
T7 polymerase‐driven inducible cell lysis for the DNA transfer from *Escherichia coli* to *Bacillus subtilis*. A. Figure shows transformation efficiency of pJScav, iBAC(cav)1 and iBAC(10) calculated from the number of colony‐forming units per μg of transformed DNA. The mean and standard errors were calculated from three replicates. B. Figure shows inhibition of the pJScav‐, iBAC(cav)1‐ and iBAC(10)‐encoded DNA transfer from *E. coli* to *B. subtilis* by addition of DNase I. *B. subtilis* cells which successfully integrated transferred DNA into the chromosome grew on chloramphenicol and emitted fluorescent light. C. Transferred DNA integrated into *amyE* locus in the *B. subtilis* chromosome. HyperLadder 1 kb (Bioline) was used as the molecular weight marker. D. Figure shows numbers of *B. subtilis* cells per ml of mixed *E. coli* and *B. subtilis* cultures, which integrated the transferred pJScav**‐**encoded DNA into their chromosomes 6 h after the induction of cell lysis. The T7 RNA polymerase‐driven inducible cell lysis system in the strain Ec(R*pT7*L123*p* *L*T7i) led to the significant increase in the number of *B. subtilis* transformants. The statistical *t*‐tests conducted to evaluate the variations in pJScav transfer confirmed the statistical difference (*P* < 0.01, indicated by star) between strains Ec(R*pT7*L123*p* *L*T7i) and Ec(R*p* *L*L123i). E. Figure depict numbers of *B. subtilis* per ml of mixed cultures, which integrated the transferred iBAC(cav)1‐ and iBAC(10)**‐**encoded DNA into their chromosomes 6 h after the induction of cell lysis. The statistical *t*‐tests to measure the differences in iBAC(cav)1 and iBAC(10) DNA transfer showed the statistical difference (indicated by the star) between the strain Ec(R*pT7*L123*pL*T7i) and Ec(R*pL*L123i).

To test the ability of pJScav, iBAC(cav)1 and iBAC(10) to integrate into the *amyE* site in the *B. subtilis* chromosome, we transformed equivalent amount of pJScav, iBAC(cav)1 and iBAC(10) DNA into the competent *B. subtilis* cells as described in the Experimental procedures. pJScav, iBAC(cav)1 and iBAC(10) did integrate into the *B. subtilis* chromosome, with pJScav yielding the highest number of transformants (7.2 × 10^4^ transformants per μg DNA; Fig. 3A).

Next, to analyse our engineered T7 RNA polymerase‐driven inducible cell lysis system, we used culture mix method (Itaya and Kaneko, 2010; Kaneko and Itaya, 2010) to transfer pJScav, iBAC(cav)1 and iBAC(10) from the lysed *E. coli* cells into *B. subtilis*. Following transformation of pJScav, iBAC(cav)1 or iBAC(10) into the *E. coli* strains Ec(Ri), Ec(R*pT7*L123*pL*T7i) and Ec(R*pL*L123i), and the temperature‐induced cell lysis, pJScav, iBAC(cav)1 and iBAC(10), DNA was taken up by *B. subtilis* cultivated in the same test tube. Sequences homologous to *amyE* target site in the *B. subtilis* chromosome flanking chloramphenicol resistance and *mVenus* genetic circuits in pJScav and iBAC(cav)1 and 10 kb DNA fragment in iBAC(10) facilitated integration of the transferred DNA into the *B. subtilis* chromosome. As a consequence, *B. subtilis* cells, which successfully integrated transferred pJScav, iBAC(cav)1 and iBAC(10) DNA into their chromosome emitted fluorescent light and grew on chloramphenicol (Fig. 3B). Furthermore, we confirmed integrations by diagnostic PCR using flanking primers and sequencing (Fig. 3C and Table S1). We verified that the DNA transfer occurred via stable DNA in the medium by the addition of DNase I, which inhibited pJScav and iBAC(cav)1 and iBAC(10)‐borne DNA transfer from *E. coli* to *B. subtilis* (Fig. 3B).

pJScav, iBAC(cav)1 and iBAC(10) transferred into the recipient *B. subtilis* with various efficiency for the donor *E. coli* strains Ec(Ri), Ec(R*pT7*L123*pL*T7i) and Ec(R*pL*L123i). When using strain Ec(Ri), the low‐efficiency transfer of pJScav and iBAC(cav)1 and iBAC(10) from *E. coli* to *B. subtilis* occurred due to spontaneous lysis of *E. coli* Ec(Ri) cells (Corchero *et al*., 2001; Juhas *et al*., 2016). Both donor strains Ec(R*pT7*L123*pL*T7i) and Ec(R*pL*L123i) transferred pJScav and iBAC(cav)1 and iBAC(10) to *B. subtilis* with higher efficiency than the strain Ec(Ri). The statistical *t*‐test analysis revealed that there was a significant difference in the transfer of pJScav, iBAC(cav) and iBAC(10) from *E. coli* to *B. subtilis* when using strains Ec(R*pL*L123i) and Ec(R*pT7*L123*pL*T7i). pJScav transfer using strain Ec(R*pT7*L123*pL*T7i) led to 112% (*P* < 0.01) increase in the number of the positive *B. subtilis* recipients when compared with the strain Ec(R*pL*L123i) 6 h after the induction of the cell lysis (Fig. 3D). Similarly, compared with the strain Ec(R*pL*L123i), iBAC(cav)1 and iBAC(10), transfer using *E. coli* strain Ec(R*pT7*L123*pL*T7i) led to 51% (*P* < 0.01) and 46% (*P* < 0.05) increase in the number of the positive *B. subtilis* recipients respectively (Fig. 3E). This showed that integration of the T7 RNA polymerase‐encoding genetic circuit into the *E. coli* chromosome in our T7 RNA polymerase‐driven inducible cell lysis system in the strain Ec(R*pT7*L123*pL*T7i) strongly improves DNA transfer from *E. coli* to *B. subtilis*.

## Conclusions

As both *E. coli* and *B. subtilis* are among the main chassis for a number of synthetic biology and biotechnology applications, it is important to develop reliable and efficient systems for the transfer of synthetic DNA (especially the high‐molecular‐weight DNA, which is prone to physical breakage) between these two cell types. The previously engineered cell lysis systems either required the chemical inducer, enzyme or mechanical force or utilized the entire prophages to disrupt the donor *E. coli* cell, or their efficiency of lysis was not very high (Itaya and Kaneko, 2010; Kaneko and Itaya, 2010; Gao *et al*., 2013; Juhas *et al*., 2016).

To address this problem, we investigated the effect of T7 RNA polymerase on the cell lysis. T7 RNA polymerase expression system is among the most efficient and widely used systems, which has been successfully applied to regulate the protein expression in a number of bacteria. We integrated three genetic circuits encoding the T7 RNA polymerase, the thermosensitive repressor and the lysis genes of three bacteriophages MS2, lambda and φX174 into the *E. coli* chromosome using streamlined lamda Red recombineering approach (Juhas *et al*., 2014a). The expression of T7 RNA polymerase in our inducible cell lysis system is regulated by the thermosensitive repressor. T7 RNA polymerase in turn regulates expression of the *pT7* promoter, which is located upstream of the bacteriophages' lysis genes. We showed that the chromosomally integrated T7 RNA polymerase significantly increases efficiency of *E. coli* cell lysis and transfer of the plasmid and BAC‐encoded DNA from the lysed *E. coli* cells into *B. subtilis*.

The chemical lysis is even faster and more efficient; however, due to the harmful and toxic effects of the phenol–chloroform, the application of the chemical lysis approach for the direct transfer of the DNA from lysed *E. coli* cells to *B. subtilis* in a single tube would be quite problematic. Furthermore, it does not address the issue of the shearing of the high‐molecular‐weight DNA.

To achieve better phage lysis, other genes crucial for the phage's life cycle could be integrated into the chromosome. It has been shown previously that only simultaneous expression of the lytic genes *lytA* and *lytH*, encoding the lysin and the holin proteins of bacteriophage ΦUS3, leads to the efficient lysis of *L. lactis*, whereas expression of holin or lysin alone does not cause significant lysis (de Ruyter *et al*., 1997). Furthermore, the genome of bacteriophage lambda has almost 50 kb and produces approximately 100 virions after each cell lysis (Gandon, 2016). Our system presented here utilizes only the single chromosomally integrated copy the phages' lysis genes. Further improvements the system should undergo therefore include integration of other genes important for the phages' life cycle. Other promoters, such as *P*_*lac*_ *‐LacI*,* P*_*mgtB*_ *, P*_*nisA*_ *, P*_*cmp*_ and the light‐inducible cI repressor can be also used to improve the efficiency of the cell lysis (Zhang *et al*., 2009; Gao *et al*., 2013; Lee *et al*., 2013; Juhas *et al*., 2016).

The possibility of utilizing other mechanisms, such as conjugative transfer of the synthetic DNA from the donor *E. coli* to the recipient *B. subtilis,* could also be investigated in more detail. Utilizing conjugative DNA transfer from *E. coli* to *Bacillus megaterium* led to the acquirement of up to 15 000 transconjugants in a single experiment (Richhardt *et al*., 2010). Although the efficiency of DNA transfer from *E. coli* to *B. megaterium* by conjugation is considerably lower than the DNA transfer from *E. coli* to *B. subtilis* presented in our study, conjugation is particularly important for other industrially relevant members of the genus with extremely poor natural competence, such as *B. megaterium*.

To our knowledge, this is the first study analysing the effect of T7 RNA polymerase on the inducible lysis of *E. coli* and DNA transfer and integration into the *B. subtilis* chromosome. Our engineered T7 RNA polymerase‐driven inducible cell lysis system has many benefits over the previously used approaches, such as no need for chemical inducer, enzyme or entire prophage, which could be detrimental to the quality of the transferred DNA. Combined with the culture mixed method (Itaya and Kaneko, 2010; Kaneko and Itaya, 2010), our system is suitable for the reliable and efficient cell lysis and DNA transfer from the lysed *E. coli* to other naturally competent hosts, such as *B. subtilis*.

## Experimental procedures

### Strains, plasmids, BACs and growth conditions

All bacterial strains, BACs and plasmids used in this study are listed in Table 1. Luria‐Bertani broth (LB) was used for routine growth of *B. subtilis* and *E. coli*. Growth media were supplemented when needed with the following concentrations of antibiotics: kanamycin (5 μg ml^−1^) and chloramphenicol (5 μg ml^−1^) for growing *B. subtilis,* and kanamycin (50 μg ml^−1^), chloramphenicol (30 μg ml^−1^) and ampicillin (100 μg ml^−1^) for growing *E. coli*. Bacterial cultures were grown at 30°C, 37°C or 42°C on solid media for 24 h. Liquid bacterial cultures were incubated on a rotatory shaker at 200 r.p.m. and 30°C, 37°C or 42°C, according to requirements. To generate competent *B. subtilis* cells, *B. subtilis* was routinely grown in a starvation medium composed of 5× minimal salts solution [potassium hydrogen phosphate (75 mg ml^−1^), potassium dihydrogen phosphate (25 mg ml^−1^), ammonium sulfate (10 mg ml^−1^), sodium citrate (1 mg ml^−1^), magnesium sulfate heptahydrate (1 mg ml^−1^)] and glucose (0.5% w/v).

**Table 1:** Bacterial strains, plasmids and bacterial artificial chromosomes (BACs) used in this study

	Characteristics	Reference
Strains		
Ec	*Escherichia coli* wild‐type strain K12 MG1655	Hayashi *et al*. (2006)
Bs	*Bacillus subtilis* wild‐type strain 168	Laboratory collection
Ec(Ri)	*E. coli*, chromosomally integr. ts repressor	Juhas *et al*. (2014a)
Ec(R*pL*L123i)	*E. coli*, integr. ts repressor, *MS2,ΦX174, λ*	Juhas *et al*. (2016)
Ec(R*pT7*L123*pL*T7i)	*E. coli*, integr. ts repressor, *MS2,ΦX174, λ* *pT7* promoter, T7 polymerase *pL* promoter	This study
Bs(acavi)	*B. subtilis*, integr. chloramphenicol, mVenus	This study
Bs(10i)	*B. subtilis*, integr. 10 kb insert	This study
Plasmids and BACs		
pSB1K3	Standard assembly plasmid, BioBricks	Parts registry
pKM208	IPTG‐inducible lambda Red system	Murphy and Campellone (2003)
pE‐FLP	FLP recombinase, constitutive expression	St‐Pierre *et al*. (2013)
pFRTKr	pSB1K3 with ts repressor	Juhas *et al*. (2014a)
pTARA	Plasmid with T7 RNA polymerase	Wycuff and Matthews (2000)
pJScav	Plasmid with chloramphenicol, mVenus	Parts registry
pFRT*pT7*L123	*MS2,ΦX174,λ* with *pT7* promoter in pSB1K3	This study
pFRT*pL*T7	T7 polymerase with *pL* promoter in pSB1K3	This study
iBAC(10)	BAC, *amyE* sites, 10 kb insert	This study
iBAC(cav)1	BAC, *amyE* sites, chloramphenicol, mVenus	This study

### Transformation of competent *B. subtilis* cells

To induce competency, *B. subtilis* was grown first in 10 ml minimal medium composed of 5× minimal salts solution, casamino acids (0.02% w/v), tryptophan (20 μg ml^−1^), iron ammonium citrate (2.2 μg ml^−1^) and glucose (0.5% w/v) at 200 r.p.m. and 37°C for 18 h; 1.4 ml of this culture was inoculated into 10 ml of the fresh minimal medium and grown at 200 r.p.m. and 37°C for another 3 h. Then, 11 ml of the starvation medium composed of 5 ×  minimal salts solution and glucose (0.5% w/v) was added and cells were incubated at 200 r.p.m. and 37°C for additional 2 h and 45 min. 0.3 ml aliquots were transformed with 15 μl of plasmid or BAC DNA in 15 ml polypropylene tubes and incubated first at 200 r.p.m. at 37°C for 1 h and then at 200 r.p.m. at 37°C for 2 h with additional 700 μl LB. Subsequently, 20–200 μl was plated onto selection plates and incubated at 37°C for 20 h. DNA integrations into the *B. subtilis* chromosome were verified by diagnostic PCR and sequencing.

### DNA amplification and modification

DNA purification was performed using Qiaquick Gel Extraction kit (Qiagen, Hilden, Germany). Plasmid isolation was performed with Qiaprep Spin Miniprep kit (Qiagen). Isolation of BACs was performed using Qiaprep Spin Miniprep kit (Qiagen) or PhasePrep BAC DNA kit (Sigma‐Aldrich, Irvine, UK) using instructions provided by the kits' manufacturer. GeneJET genomic DNA purification kit (Thermo Scientific, Loughborough, UK) was used to isolate genomic DNA from *B. subtilis*. DNA sequencing was performed by Source Bioscience. Oligonucleotide primers for PCR amplification were synthesized by Sigma‐Aldrich and the longer DNA fragments by Integrated DNA Technologies (IDT). PCR amplification of DNA fragments was conducted in a 25 μl or 50 μl final reaction volume with Dream Taq master mix kit (Thermo Scientific), Phusion DNA polymerase (Thermo Scientific) or Q5 high‐fidelity DNA polymerase (NEB) using instructions recommended by the supplier. Assembly of DNA fragments was performed using Gibson Isothermal Assembly in a 5.2 μl final reaction volume (Gibson *et al*., 2009; Merryman and Gibson, 2012; Juhas *et al*., 2014a). Confirmation of the correctly assembled DNA fragments was performed by diagnostic PCR using flanking primers and DNA sequencing.

### DNA integration into the *E. coli* chromosome

The chemically competent *E. coli* and the electro‐competent *E. coli* were generated using the modified Hanahan *et al*. (1991) and Miller and Nickoloff (1995) methods respectively. Lambda Red recombineering to integrate DNA into the *E. coli* chromosome was performed using the previously described procedure (Juhas *et al*., 2014a; Juhas and Ajioka, 2015a,2015b). Briefly, plasmid pKM208 encoding IPTG‐inducible lambda Red recombinase system was electroporated into *E. coli*. The cells were selected on the ampicillin plates and then grown in the liquid LB with ampicillin at 30°C overnight, diluted (1:100 dilution) into fresh LB with ampicillin and grown at 30°C to OD_600_ 0.2. After the addition of IPTG (1 mM) at OD_600_ 0.2, the bacterial culture was grown to OD_600_ 0.5. Cells were washed twice and then resuspended in 10% glycerol and electroporated with 100 μl of the amplified DNA fragments harbouring sequences homologous to the integration sites in the *E. coli* chromosome. Transformants with integrated DNA were selected by growth on selective plates at 30°C overnight. pKM208 was cured out by growth at 42°C. Integration of DNA fragments into the *E. coli* chromosome was verified by diagnostic PCR and sequencing. The kanamycin resistance marker was flipped out from the chromosome using the thermosensitive plasmid pEP‐FLP encoding constitutively expressed flippase (FLP) recombinase (St‐Pierre *et al*., 2013).

### DNA transfer from *E. coli* to *B. subtilis*

DNA transfer from *E. coli* to *B. subtilis* was performed using the modified culture mix method (Itaya and Kaneko, 2010; Kaneko and Itaya, 2010) described previously (Juhas *et al*., 2016). Briefly, *E. coli* grown in liquid LB medium at 30°C for 24 h was diluted (1:200 dilution) into a fresh 20 ml LB with appropriate antibiotics and grown at 30°C for 5 h. Cells were centrifuged at 5000 r.p.m. for 8 min and then resuspended in a fresh 20 ml LB and grown for 1 h. *B. subtilis* grown in a liquid LB at 37°C for 17 h was diluted (1:200 dilution) into a 20 ml TFI medium [5 × minimal salts solution, glucose (0.5% w/v), casamino acids (2% w/v), tryptophan (50 μg ml^−1^), threonine (50 μg ml^−1^), leucine (50 μg ml^−1^), arginine (50 μg ml^−1^)] and cultivated at 37°C for 5 h. *E. coli* and *B. subtilis* cells were mixed (1:1 ratio) and grown at 42°C for 6 h. Then, 200 μl of the mixed bacterial culture was transferred on LB plate supplemented with chloramphenicol. The remaining *E. coli* cells were eliminated by inducing *B. subtilis* sporulation and heat treatment. First, colonies scraped off the plates were resuspended in 1 ml of 2 × SG medium [Difco nutrient broth (Difco Laboratories, Detroit, MI, USA; 16 mg ml^−1^), glucose (0.1% w/v), 1 mM FeSO_4_ (0.1% v/v), 0.1 M MnCl_2_.4H_2_O (0.1% v/v), KCl (2 mg ml^−1^), MgSO_4_.7H_2_O (0.5 mg ml^−1^), 1 M Ca(NO_3_)_2_ (0.1% v/v)] and grown on SG plates for 72 h. Then, cells scraped off the 2 × SG plates were washed with water and incubated at 90°C for 10 min. Subsequently, 100 μl was transferred on LB plates supplemented with chloramphenicol and grown at 37°C overnight. The CFUs and standard deviations were calculated from three biological replicates.

### Bacterial viability and growth assays

To quantify the viability and growth of the bacterial strains, several assays were performed.

In the first assay, the absorbance of the bacterial cultures was measured using 96‐well microplates (clear, Sterilin Sero‐Well, Loughborough, UK). Overnight, bacterial cultures were normalized to OD_600_ of 0.05 and 200 μl of the normalized cultures was transferred into the microplate wells. Microplates were incubated in the microplate reader (Fluostar Omega, BMG Labtech, Aylesbury, UK) at 30°C for 3 h and at 42°C for another 21 h or at 30°C for 24 h. Absorbance was quantified using the automatically repeated protocol (double orbital shaking at 500 r.p.m., cycle time 60 min, absorbance filter 600 nm). The average absorbance and the standard deviations were calculated from three experiments.

In the second assay, the overnight bacterial cultures adjusted to optical density OD_600_ of 0.05 were incubated at 30°C for 3 h and then at 42°C for another 6 h on a rotatory shaker at 200 r.p.m. Bacterial cultures were serially diluted up to 10^−6^ in a fresh LB media (pre‐heated to 42°C) 6 h after the induction of cell lysis; 100 μl of the diluted bacterial cultures was spread on solid LB media plates pre‐heated to 42°C and incubated overnight at 42°C. The average numbers of CFUs and standard errors were calculated from three independent biological replicates.

In the third assay, the viability of the *E. coli* cells was analysed using the Live/Dead BacLight™ bacterial viability kit (Lifetechnologies, Paislay, UK), according to supplier's instructions. First, bacterial cells were incubated on a rotatory shaker at 200 r.p.m. and 30°C for 6 h and then at 42°C for another 6 h. Bacterial cultures were washed with 0.85% NaCl and adjusted to 1 × 10^8^ bacteria per 1 ml of 0.85% NaCl. Bacteria were then mixed with the red‐fluorescent nucleic acid stain propidium iodide and the green‐fluorescent nucleic acid stain SYTO9 (3 μl of stain per 1 ml of medium containing bacteria) and examined by fluorescent microscopy (Nikon Microphot‐SA, Kingston Upon Thames, Surrey, UK). The numbers of the dead bacteria (propidium iodide‐stained) and the live bacteria (SYTO‐9 stained) were calculated 6 h after the induction of the cell lysis. The means and standard errors were calculated from three biological replicates.

### Chemical lysis of *E. coli*

The control chemical lysis of *E. coli* cells was performed by the modified method (Song *et al*., 1999), described earlier. Briefly, bacterial cultures were serially diluted up to 10^−6^ in LB media and 500 μl thereof was centrifuged at 8000 r.p.m. for 5 min. The pellets were resuspended in 500 μl of the 1:1 of phenol and chloroform solution (v/v). Then, the samples were mixed vigorously by vortexing for 10 s, incubated with shaking at room temperature for 30 min and centrifuged at 13 000 r.p.m for 5 min. The pellets were resuspended in 100 μl of a fresh LB media, spread on solid LB media plates and incubated overnight at 37°C. The mean CFU values and standard errors were calculated from three replicates.

### Sequence analyses

DNA sequences were analysed using tblastx, blastn (Altschul *et al*., 1990) and position‐specific iterated blast (psi‐blast; Altschul *et al*., 1997) algorithms interrogating the National Centre for Biotechnology Information (NCBI) website (http://ncbi.nlm.nih.gov). The *E. coli* genome sequence was obtained from the *E. coli* K‐12 project website (http://www.xbase.ac.uk/genome/escherichia-coli-str-k-12-substr-mg1655). The *B. subtilis* genome sequence was obtained from the BioCyc (http://bsubcyc.org/) and SubtiWiki (http://subtiwiki.uni-goettingen.de/) databases. The sequences of the parts (promoters, RBS, terminators) of the DNA constructs were obtained from the Registry of Standard Biological Parts (http://parts.igem.org/Main_Page), the Addgene non‐profit plasmid repository (http://www.addgene.org/) and the NCBI website. DNA sequencing was performed by Source Bioscience.

## Conflict of interest

There is no conflict of interests related to this manuscript.

## Supporting information


**Fig. S1.** Genetic circuits *pT7*L123, Repr‐ts‐1 and *pL*T7pol. Figure shows schematic view of the genetic circuits *pT7*L123, Repr‐ts‐1 and *pL*T7pol, which were integrated into the *E. coli* chromosome to generate the T7 RNA polymerase‐driven inducible cell lysis system.

**Table S1.** Primers used in this study.

**Data S1.** Plasmids pFRT*pT7*L123 and pFRT*pL*T7. Sequence of plasmids pFRT*pT7*L123 and pFRT*pL*T7 harbouring genetic circuits *pT7*L123 and pLT7pol (highlighted green) respectively.
