# Supplementary figures and images for: T7 RNA polymerase‐driven inducible cell lysis for DNA transfer from Escherichia coli to Bacillus subtilis
Source: Microb Biotechnol. 2017 Aug 16;10(6):1797–808. doi: 10.1111/1751-7915.12843 (PMC5658589; doi:10.1111/1751-7915.12843)

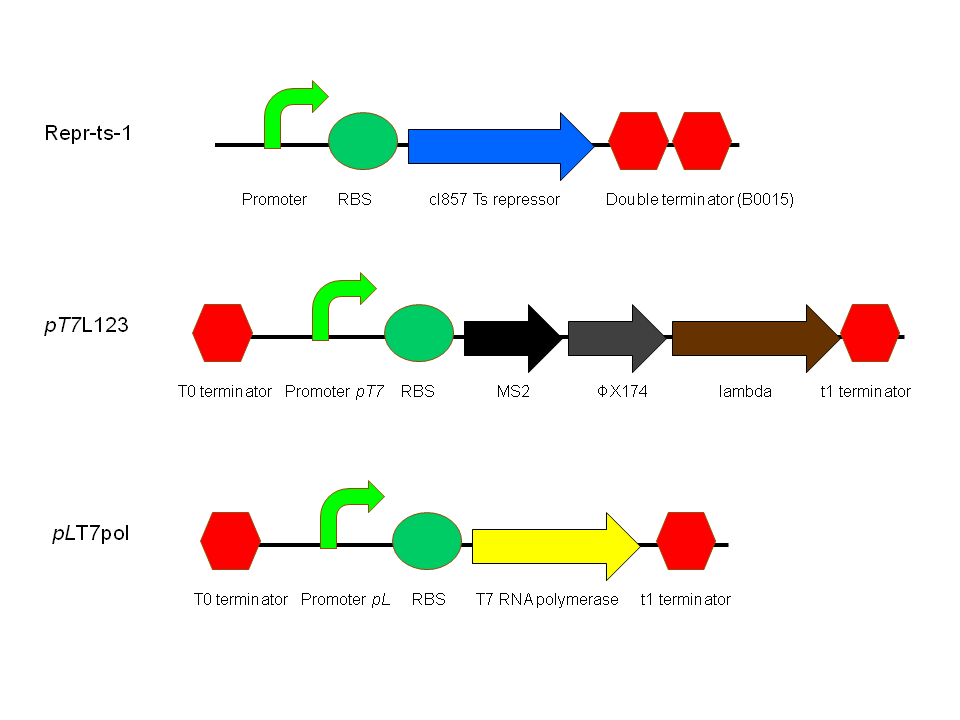

Supplement: Supplementary file 1 — Fig. S1. Genetic circuits pT7L123, Repr‐ts‐1 and pLT7pol. Figure shows schematic view of the genetic circuits pT7L123, Repr‐ts‐1 and pLT7pol, which were integrated into the E. coli chromosome to generate the T7 RNA polymerase‐driven inducible cell lysis system. [file MBT2-10-1797-s001.tiff]
