# Supplementary material for: T7 RNA polymerase‐driven inducible cell lysis for DNA transfer from Escherichia coli to Bacillus subtilis
Source: Microb Biotechnol. 2017 Aug 16;10(6):1797–808. doi: 10.1111/1751-7915.12843 (PMC5658589; doi:10.1111/1751-7915.12843)
Supplement: Supplementary file 2 — Table S1. Primers used in this study. [file MBT2-10-1797-s002.doc]

**Supplementary Table 1. Primers used in this study.**

**Primer (Sequence 5’→ 3’)**

REPR-IF: Repr-ts-1 integration primer forward

TTATGCAGGACAACAATCAGCAAGGCAAAGGCGTCTCTTTGTCAAAAGCGATCAACATCATTGAGAGTATCACGAGGCAGAATTTCAGAT

REPR-IR: Repr-ts-1 integration primer reverse

AATATTGCGCATTAATGCTTCCACTTCTTCGACTGCGGAGACATCGTTGCGTAAATTGGCTTCGGTTTTAAAGAAAAAGGGCAGG

L123-IF: *pT7*L123 integration primer forward

GAAATGCGTCGCGGATGTCAGCAGGCGGAAGAACAGCTCAAAATGCTGATTGATTATCAGAATGAAGTATCACGAGGCAGAATTTCAGAT

L123-IR: *pT7*L123 integration primer reverse

TGCAAACGTTGTTTTTTTTCTCGCCAACTGTTCAGGGCAATGTCAACTTTCTGCGTCCACTGATTACGGTTTTAAAGAAAAAGGGCAGG

T7-IF: *pL*T7pol integration primer forward

CTCCAAATACACCAAAGCAATGTATATGGATCTGCTGGCTCTGCTTTATCGGTTGATGGCGAAATCGTATCACGAGGCAGAATTTCAGAT

T7-IR: *pL*T7pol integration primer reverse

TTTCTGACGTAAAACAGTCGCTAATGGGGAAATAAATCCGTAAGCCAATAAAATGCCGAGGAAAGTCGGTTTTAAAGAAAAAGGGCAGG

amy-TF: *amyE* integration test primer forward

CCAGTCTTCACATCGGTTTGAAAGG

amy-TR: *amyE* integration test primer reverse

ATCATTGATGGTTTCTTTCGGTAAGTCC

pT7-1: pFRT*pL*T7 Gibson Isothermal Assembly primer 1

GGCGGTGATACTGAGCACAATTAAAGAGGAGAAAATGAACACGATTAACATCGCTAAGAACGAC

pT7-2: pFRT*pL*T7 Gibson Isothermal Assembly primer 2

GTCGTTCTTAGCGATGTTAATCGTGTTCATTTTCTCCTCTTTAATTGTGCTCAGTATCACCGCC

pT7-3: pFRT*pL*T7 Gibson Isothermal Assembly primer 3

TCGGACTTCGCGTTCGCGTAACTGTAACAGAGCATTAGCGCAAGGTGATTTTTG

pT7-4: pFRT*pL*T7 Gibson Isothermal Assembly primer 4

CAAAAATCACCTTGCGCTAATGCTCTGTTACAGTTACGCGAACGCGAAGTCCGA

pL123-1: pFRT*pT7*L123 Gibson Isothermal Assembly primer 1

TAATACGACTCACTATAGGGAATACAAGCTACTTGTTCTTTTTGCAATTAAAGAGGAGAAAATGGA

pL123-2: pFRT*pT7*L123 Gibson Isothermal Assembly primer 2

AGGTATTAGAAGAATATCCTGATTCAGGTGAAAATATTGTTGATGCGCTGGCAGT

pL123-3: pFRT*pT7*L123 Gibson Isothermal Assembly primer 3

ACTGCCAGCGCATCAACAATATTTTCACCTGAATCAGGATATTCTTCTAATACCT

pL123-4: pFRT*pT7*L123 Gibson Isothermal Assembly primer 4

TGCAAAAAGAACAAGTAGCTTGTATTCCCTATAGTGAGTCGTATTATGGACTCACAAAGAAAAAAC
